# Association between Socioeconomic Status and One-Month Mortality after Surgery in 20 Primary Solid Tumors: a Pan-Cancer Analysis

**DOI:** 10.7150/jca.46088

**Published:** 2020-07-11

**Authors:** Wei Sun, Huaqiang Zhou, Minghua Cheng, Shaohui Zhuang, Zeting Qiu

**Affiliations:** 1 Department of Anesthesiology, The First Affiliated Hospital of Shantou University Medical College, Shantou, Guangdong, People's Republic of China.; 2 Department of Medical Oncology, Sun Yat-sen University Cancer Center, Guangzhou, Guangdong, People's Republic of China.

**Keywords:** Socioeconomic status, One-month postoperative mortality, Solid tumor, Pan-cancer analysis

## Abstract

**Background:** Surgery is the main therapy for primary solid tumors. One-month postoperative mortality remains an important criterion for assessing the quality of surgery. Socioeconomic status (SES) plays an important role in the biopsychosocial medical model. We performed a pan-cancer analysis to explore the relationship between SES and one-month mortality after surgery in 20 primary solid tumors.

**Methods:** Eight SES factors and the top 20 common cancer sites were selected between 2007 and 2014 based on the Surveillance, Epidemiology, and End Results database. The primary outcome was that patients died within one month after surgery. The control group survived beyond one month. Multivariable logistic regression model, propensity score matching and subgroup analysis were used to detect the association.

**Results:** There were 15980 (1.4%) patients who died within one month after surgery among 1132666 patients with primary solid cancers. Patients with unmarried status (aOR 1.516, 95% CI 1.462-1.573, *P* < 0.001), Medicaid/uninsured status (aOR 1.610, 95% CI 1.534-1.689, *P* < 0.001), low income (aOR 1.122, 95% CI 1.053-1.196, *P* < 0.001), low education (aOR 1.088, 95% CI 1.033-1.146, *P* = 0.001), or high poverty (aOR 1.085, 95% CI 1.026-1.147, *P* = 0.004) had high risks of one-month postoperative mortality. After propensity score matching and subgroup analysis, the effects of marriage and insurance on mortality were almost consistent with overall.

**Conclusions:** There was a strong association between SES status and one-month postoperative mortality in primary solid tumors. Socioeconomically disadvantaged people had high risks of dying within one month after surgery. Unmarried or Medicaid/uninsured status were associated with much higher risks than other factors.

## Introduction

Cancer has been one of the greatest enemies of humanity. It was estimated that there were 18.1 million new cancer cases and 9.6 million cancer deaths worldwide in 2018 1. Cancer is commonly classified into two broad types, solid tumor cancers and hematological cancers, and solid tumors make up most of cancers. Cancer-directed surgery is the main therapy for solid tumors, and it is almost the only solution to cure solid tumors. Although patients chose cancer-directed surgery for life extension, 0.5% of patients developed at least one postoperative complication and died in a few of weeks after elective surgery 2. The situation was more severe in patients receiving emergency abdominal surgery, of which the overall mortality rate increased to 5.4% by 30 days 3. One-month postoperative mortality represents that patients' die within one month since surgery. One-month mortality after surgery remains an important criterion when evaluating the quality of surgical treatment 4. It is also an important indicator of short-term survival after surgery.

Compared to the traditional biomedical model, the biopsychosocial medical model emphasizes the important role of socioeconomic status (SES) in health care services, such as insurance status, marital status and poverty level 5, 6. Disparities in SES have caused concern in health care system, and socioeconomic factors have been proven to affect prognosis in different cancers 7. Socioeconomically disadvantaged people tend to have worse survival outcomes when compared to socioeconomically advantaged people 8, 9. However, few studies focus on the association between SES and one-month postoperative mortality in primary solid tumors.

In this study, we made use of the Surveillance, Epidemiology, and End Results (SEER) database from the United States, and performed a pan-cancer analysis to explore the relationship between socioeconomic factors and one-month mortality after cancer-directed surgery in 20 primary solid tumors. The objective of this study was to assess whether SES influenced one-month postoperative mortality of solid tumors in a retrospective SEER population-based cohort.

## Materials and Methods

### Data sources

The SEER database composes of 18 cancer registries and covers approximately 30% of the population in the United State 10. We retrieved patient data through the SEERStat software (version 8.3.5, released on March 6, 2018, Authorization number: 12738-Nov.2016). The data in the SEER database were de-identified, and approval for this study was waived by the local ethics committee. All the data are available in the SEER database.

### Inclusion and exclusion criteria

Patients undergoing surgery for primary solid tumors were identified during 2007 and 2014. We chose the top 20 most common primary solid tumor site in the United States, classified as bladder, brain, breast, cervix, colorectum, esophagus, kidney, larynx, liver, lung, melanoma, oral cavity, ovary, pancreas, prostate, small intestine, stomach, testis, thyroid, and uterine. Inclusion criteria: (1) with primary solid tumors; (2) undergoing any cancer-directed surgery; (3) diagnosed between 2007 and 2014 (because insurance variable was missing before 2007). Exclusion criteria: (1) unknown or missing variables; (2) with autopsy or death certificate; (3) multiple primary tumors; (4) unknown follow-up time and events.

### Variable selection

We collected the following data from the SEER database: demographics factors (age at diagnosis, gender), socioeconomic factors (contained race, marital status, insurance status, income level, education level, residence, unemployment level, poverty level) and clinicopathological factors (cancer site, SEER stage, surgical therapy, causes of death and survival months). Age group was grouped as <50, 50-59, 60-69 and >69 years old (according to the median, first and third quartiles). Gender was classified as male and female. Race was classified as minority and non-Hispanic white (NHW). Marriage was classified as married and unmarried (including divorced, separated, single or widowed patients). Insurance was classified as insured and Medicaid/uninsured. Levels of income, education, unemployment and poverty were classified as the top 50% and the bottom 50% according to the median. Residence was classified as metropolitan and rural. SEER stage was classified as localized, regional and distant stages. Patients with unknown categories of any variables were excluded.

### Statistical analysis

All continuous variables followed a normal distribution, thus continuous variables were described as mean and standard deviation. Categorical variables were described as frequencies and percentages. The primary outcome was that patients died within one month after surgery. The control group survived beyond one month after surgery 11. Pearson's chi-squared test was used to detect statistical significance between categorical variables and categorical variables. We used the multivariable logistic regression model to detect associations between SES and one-month mortality. The model was adjusted for age at diagnosis, gender and SEER stage. The greater the odds ratio value, the greater the possibility of dying within one month after surgery. Subgroup analysis by cancer site, gender, age group and SEER stage was also performed. Due to disequilibrium between groups, we performed propensity score matching (PSM) with a ratio of 1:1 by R packages of MatchIt. All statistical analysis was done by R software (version 3.6.2, released on February 29, 2020). Two-sided *P* values < 0.05 was considered significant.

## Results

### Baseline characteristics of included patients

Figure S1 showed the screening process. We reviewed 5017764 patients undergoing cancer-directed surgery from the SEER database. According to the inclusion and exclusion criteria, we finally enrolled 1132666 patients undergoing surgery for primary solid tumors during 2007 and 2014 from the SEER database for the following analysis. Table 1 showed that there were 15980 (1.4%) patients who died within one month after surgery among these patients. Generally, patients with breast cancer (301471, 26.6%), colorectal cancer (161466, 14.3%) and prostate cancer (136515, 12.1%) occupied the most. The majority of patients were female (671020, 59.2%), NHW (805053, 71.1%), insured (988967, 87.3%) and metropolitan (1008373, 89.0%). Patients' surviving beyond one month after surgery tended to be younger (60.1 ± 14.7 years old) and with localized stages (592017, 53.0%). Table S1 showed the baseline characteristics of the included patients by cancer sites. Patients with brain cancer (6.5%), stomach cancer (4.0%) and colorectal cancer (3.8%) had higher unadjusted crude one-month postoperative mortalities, and patients with breast cancer (0.1%), prostate cancer (0.2%) and thyroid cancer (0.2%) had lower crude one-month postoperative mortalities.

### Multivariable logistic regression analysis as a whole

**Figure 1:**
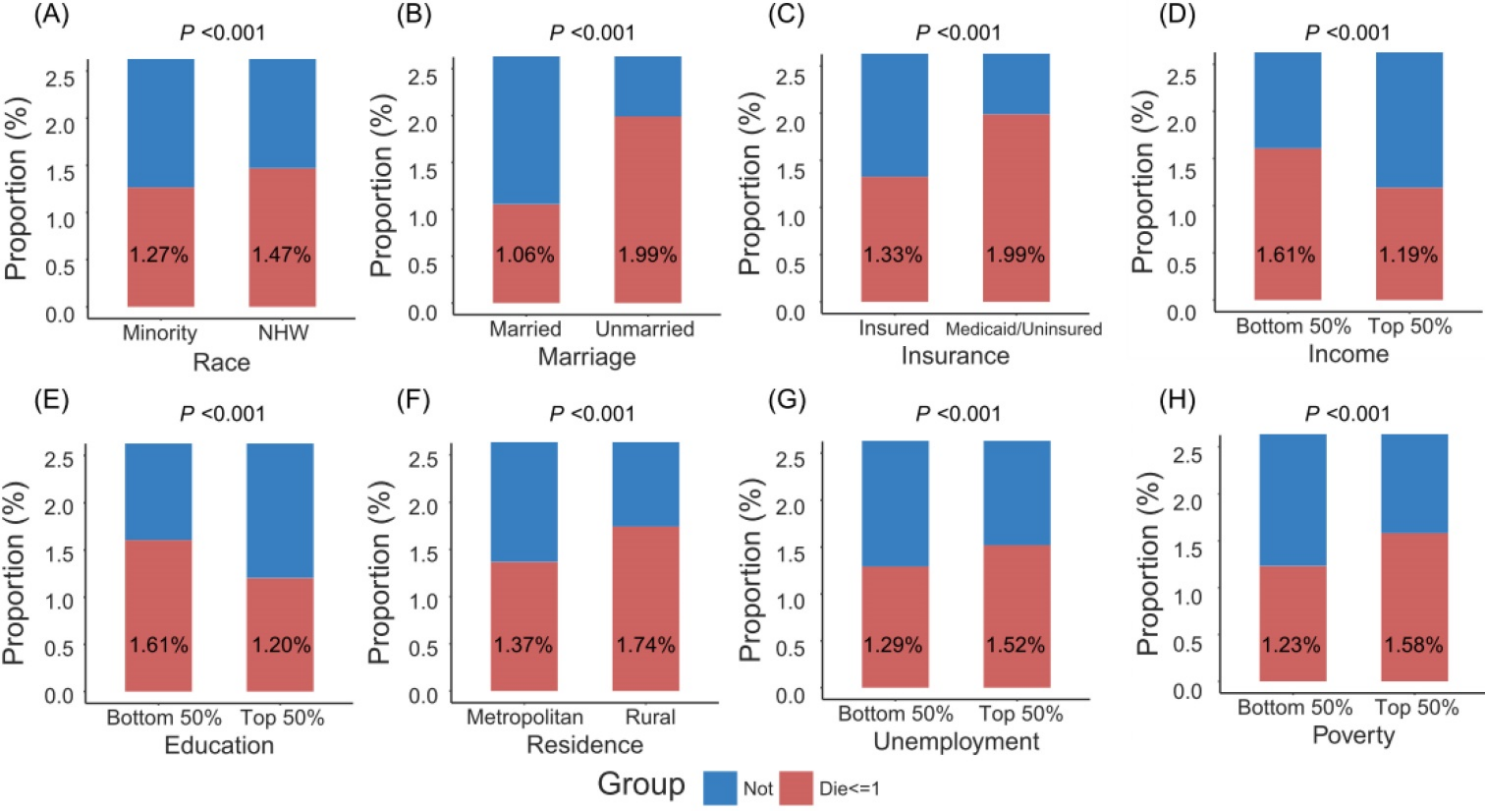
The proportion of patients dying within one month after surgery by socioeconomic factors. Abbreviations: NHW, non-Hispanic white.

Figure 1 showed that patients with minority, unmarried status, Medicaid/uninsured status, low income, low education, rural residence, high unemployment and high poverty were more likely to die within one month after surgery. More remarkably, unmarried patients had higher crude one-month postoperative mortality than married patients (2.0% versus 1.1%), and Medicaid/uninsured patients had higher crude one-month postoperative mortality than insured patients (2.0% versus 1.3%). As shown in Table 2, adjusted by age (per one year), gender and SEER stage in the multivariate logistic model, those with unmarried status (adjusted odds ratio [aOR] 1.516, 95% confidence interval [CI] 1.462-1.573, *P* < 0.001), Medicaid/uninsured status (aOR 1.610, 95% CI 1.534-1.689, *P* < 0.001), low income (aOR 1.122, 95% CI 1.053-1.196, *P* < 0.001), low education (aOR 1.088, 95% CI 1.033-1.146, *P* = 0.001), or high poverty (aOR 1.085, 95% CI 1.026-1.147, *P* = 0.004), had significantly high risks of one-month mortality after surgery. No significant association was found between one-month postoperative mortality and race, residence, unemployment. After adjustment of PSM, no statistical significances were detected between two groups for cancer site, age, gender and SEER stage (All *P* values > 0.900, Table S2). The multivariate logistic model still found that patients with unmarried status (aOR 1.333, 95% CI 1.265-1.403, *P* < 0.001) and Medicaid/uninsured status (aOR 1.424, 95% CI 1.320-1.536, *P* < 0.001) were more likely to die within one month after surgery (Table S3).

### Subgroup analysis by cancer site, gender, age group and SEER stage

**Figure 2:**
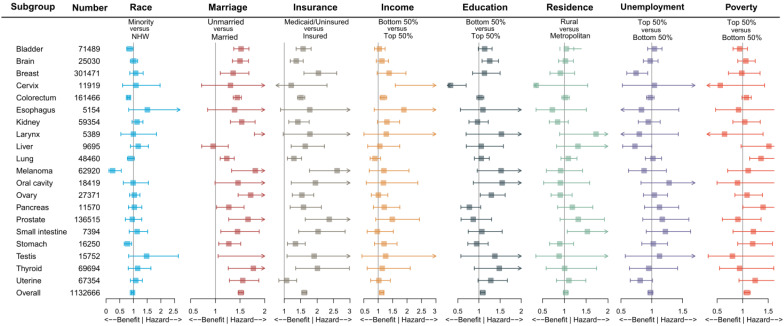
Impact of different socioeconomic factors on one-month mortality after surgery by cancer sites. Note: The aORs were adjusted by age (per one year), gender (male or female), SEER stage (localized, regional or distant), except for brain and larynx by age and gender, for prostate by age, for cervix, ovary, testis and uterine by age and SEER stage; Box indicated aOR; Segment indicated 95% confidence interval; aOR smaller than 1 indicated benefit, and aOR larger than 1 indicated hazard. Abbreviations: NHW, non-Hispanic white; aOR, adjusted odds ratio.

To further reduce the impact of cancer site, gender, age group and SEER stage on the outcome, we performed subgroup analysis. Figure 2 and Table S4 demonstrated the impact of socioeconomic factors on one-month mortality after surgery in different cancer site subgroups. It was found that unmarried and Medicaid/uninsured patients were still more likely to die within one month after surgery. Low-income patients with cervical cancer (aOR 4.157, 95% CI 1.595-10.839, *P* = 0.004) and colorectal cancer (aOR 1.173, 95% CI 1.064-1.294, *P* = 0.001) had high risks of one-month postoperative mortality, low-education patients with brain cancer (aOR 1.253, 95% CI 1.073-1.464, *P* = 0.004) had high risks of one-month postoperative mortality, and high-poverty patients with lung cancer (aOR 1.361 95% CI 1.137-1.629, *P* < 0.001) had high risks of one-month postoperative mortality. Table S5, Table S6 and Table S7 showed the subgroup analysis by gender, age group and SEER stage. We found that the effects of marriage and insurance on one-month mortality were almost consistent with overall.

## Discussions

In this study, we included 1132666 patients' undergoing cancer-directed surgery for 20 primary solid tumors during 2007 and 2014 from the SEER database in the United States, and detected an association between eight socioeconomic factors and one-month mortality after surgery. Among the population, 15980 (1.4%) patients died within one month after surgery. After adjustment of multivariate logistic analysis, SES of unmarried status, Medicaid/uninsured status, low income level, low education level, and high poverty level had significantly high risks of one-month mortality after surgery. Unmarried status and Medicaid/uninsured had the greatest impact on one-month mortality. To reduce the influence of confounding variables containing cancer site, gender, age group and SEER stage, we also performed PSM re-grouping and subgroup analysis. As a result, the effects of marriage and insurance on one-month mortality after surgery kept the same.

Marital status and insurance status played a remarkable role in one-month postoperative mortality, whether in overall analysis or subgroup analysis. Plenty of studies have proved the influence of these two factors on long-term prognosis of cancers 12. Unmarried patients, especially widowed patients, were significantly associated with higher risks of death compared with married patients. Unmarried patients usually had a high incidence of depression, as well as less social supports from spouses and family, and suffered mental stress during the perioperative period 13. When compared with Medicaid or uninsured patients, insured patients tended to have early cancer stage at diagnosis, and were more likely to undergo treatment, thus with better prognosis 14. Uninsured patients often failed to pay high medical bills, losing the opportunity for timely screening and treatment 15. We used a popular method of PSM to create a balanced covariate distribution between two groups, and the effect of marriage and insurance remained.

Our study was a comprehensive update of a prior study 11, which identified determinants of one-month mortality after surgery. Besides married and insured status, we also found that low-income, low-education and high-poverty patients with primary solid tumors were more likely to die within one month after surgery in the multivariable analysis. It is important to note that, after balance of PSM, the effect of education and poverty became insignificant. Cancer site subgroup analysis demonstrated that the high risk of low education was mainly attributed to the brain cancer subgroup , while the high risk of high poverty was mainly attributed to the lung cancer subgroup. Mahal's research identified non-white as a high-risk factor for death within one month after surgery 11, but this effect disappeared in our study, and non-white even became a protective factor after PSM. Subgroup analysis showed that the protective effect of minority came from patients aged more than 69 years old and with SEER distant stage. Our results suggested that the impacts of socioeconomic factors on one-month postoperative mortality varied by subgroup.

The influence of psychosocial factors on diagnosis, treatment and prognosis in cancer patients has caused widespread concern 16, 17. In this study, we concentrated on SES, and found that there was a strong association between SES and short-term surgical outcomes after surgery. Especially in patients with unmarried or Medicaid/uninsured status, the odds of one-month mortality were much higher than other factors. The potential mechanism might lie in that, patients with socioeconomic advantages are more likely to obtain sufficient family support and great financial security. These patients are always accompanied by adequate financial circumstances and optimal plans from medical experts. Improving SES could reduce one-month postoperative mortality. According to the biopsychosocial medical model, medical care providers should strengthen mental support for unmarried patients besides providing medical care 6, 18. It's the government's responsibility to expand wider insurance coverage for uninsured patients. If we improve the SES for the disadvantaged ones with solid tumors, their short-term survival after surgery will improve.

The results of our study, which found that socioeconomically disadvantaged people with primary solid tumors were at a high risk of death within one month after surgery, were in line with the prior study 11. However, compared to the prior study, our study involved a larger sample size and a longer period with up-to-date information from 2007 to 2014. Moreover, our current study, taking eight socioeconomic factors and 20 solid tumors into account, was more broadly considered than the prior one. For clinical features of included patients, Mahal's study chose AJCC tumor stage, which described the size of the primary tumor and any spread of the primary tumor into adjacent tissue 19. We used clinical SEER stage provided by the database to better represent the whole extent of spread of cancer generally.

Several limitations should be addressed in the current study. Firstly, some other influencing factors, such as concomitant diseases, postoperative complications or eating habits, might affect one-month mortalities of patients with primary solid tumors. These influencing factors should be taken into account for further studies. However, it's a pity that the current SEER database does not provide data about these factors 2, 20. Secondly, the method we calculated one-month mortality remained to be discussed. The follow-up time provided by the SEER database was recorded in months instead of in days. It might cause calculation problems. For example, if a patient was diagnosed at the beginning of one month and died after surgery at the end of the following month, the follow-up time would be recorded as one month in the SEER database. However, the patient died the second month after surgery actually. In any case, the one-month mortality defined in our study still represented the postoperative short-term prognosis. Thirdly, when it came to some socioeconomic factors, containing household income, education level, residence, unemployment and poverty, the SES information was not at patient-level, but at regional level. We just simply divided them into the top and bottom 50% according to the median. In this regard, the impact of patient-level personal data on primary outcomes may be more meaningful. Fourthly, given that it was a retrospective cohort study, our analysis could only reveal correlations between SES and one-month postoperative mortality. So it is worth exploring whether there is an underlying causality relationship. More studies are necessary to confirm the result.

In conclusion, there was a strong relationship between SES and one-month postoperative mortality in primary solid tumors. Socioeconomically disadvantaged people tended to have high risks of one-month mortality after surgery. Especially in patients with unmarried or Medicaid/uninsured status, the risks were much higher than other factors. It needs to be further evaluated in more clinical trials.

## Supplementary Material

Supplementary figure and tables.

## Figures and Tables

**Table 1:** Baseline characteristics of included patients undergoing surgery for 20 primary solid tumors

Characteristic	Die within one month	Survive beyond one month	Characteristic	Die within one month	Survive beyond one month
	(N=15980)	(N=1116686)		(N=15980)	(N=1116686)
**Tumor site**			**Age**	72.9 ± 14.0	60.1 ± 14.7
Bladder	1945 (12.2)	69544 (6.2)	**Race**		
Brain	1634 (10.2)	23396 (2.1)	NHW	11832 (74.0)	793221 (71.0)
Breast	432 (2.7)	301039 (27.0)	Minority	4148 (26.0)	323465 (29.0)
Cervix	55 (0.3)	11864 (1.1)	**Marriage**		
Colorectum	6211 (38.9)	155255 (13.9)	Married	7420 (46.4)	695364 (62.3)
Esophagus	75 (0.5)	5079 (0.5)	Unmarried	8560 (53.6)	421322 (37.7)
Kidney	664 (4.2)	58690 (5.3)	**Insurance**		
Larynx	61 (0.4)	5328 (0.5)	Insured	13121 (82.1)	975846 (87.4)
Liver	238 (1.5)	9457 (0.8)	Medicaid/Uninsured	2859 (17.9)	140840 (12.6)
Lung	1353 (8.5)	47107 (4.2)	**Income**		
Melanoma	191 (1.2)	62729 (5.6)	Top 50%	6422 (40.2)	532110 (47.7)
Oral cavity	119 (0.7)	18300 (1.6)	Bottom 50%	9558 (59.8)	584576 (52.3)
Ovary	707 (4.4)	26664 (2.4)	**Education**		
Pancreas	395 (2.5)	11175 (1.0)	Top 50%	6640 (41.6)	544476 (48.8)
Prostate	245 (1.5)	136270 (12.2)	Bottom 50%	9340 (58.4)	572210 (51.2)
Small intestine	280 (1.8)	7114 (0.6)	**Residence**		
Stomach	656 (4.1)	15594 (1.4)	Metropolitan	13812 (86.4)	994561 (89.1)
Testis	60 (0.4)	15692 (1.4)	Rural	2168 (13.6)	122125 (10.9)
Thyroid	159 (1.0)	69535 (6.2)	**Unemployment**		
Uterine	500 (3.1)	66854 (6.0)	Top 50%	8922 (55.8)	577853 (51.7)
**Gender**			Bottom 50%	7058 (44.2)	538833 (48.3)
Male	8211 (51.4)	453435 (40.6)	**Poverty**		
Female	7769 (48.6)	663251 (59.4)	Top 50%	9134 (57.2)	568072 (50.9)
**Age group**			Bottom 50%	6846 (42.8)	548614 (49.1)
<50	861 (5.4)	238015 (21.3)	**SEER stage**		
50-59	1725 (10.8)	276960 (24.8)	Localized	3845 (24.1)	592017 (53.0)
60-69	3105 (19.4)	311907 (27.9)	Regional	4952 (31.0)	280494 (25.1)
>69	10289 (53.6)	289804 (26.0)	Distant	5286 (33.1)	80874 (7.2)

Note: Due to uncommon classification of SEER stage, cancer sites of brain, larynx and prostate were not considered for characteristic of SEER stage. All variables were expressed as frequency (percent), except for age as mean ± standard deviation; Abbreviations: N, number; NHW, non-Hispanic white; SEER, Surveillance Epidemiology, and End Results.

**Table 2:** Multivariate logistic regression analysis of association between socioeconomic factors and one-month postoperative mortality

Characteristic	aOR	95% CI	*P*
**Race (versus NHW)**			
Minority	0.965	0.926-1.005	0.087
**Marriage (versus Married)**			
Unmarried	1.516	1.462-1.573	<0.001
**Insurance (versus Insured)**			
Medicaid/Uninsured	1.610	1.534-1.689	<0.001
**Income (versus Top 50%)**			
Bottom 50%	1.122	1.053-1.196	<0.001
**Education (versus Top 50%)**			
Bottom 50%	1.088	1.033-1.146	0.001
**Residence (versus Metropolitan)**			
Rural	1.031	0.976-1.089	0.276
**Unemployment (versus Bottom 50%)**			
Top 50%	0.977	0.937-1.018	0.264
**Poverty (versus Bottom 50%)**			
Top 50%	1.085	1.026-1.147	0.004

Note: The ORs were adjusted by age (per one year), gender (male or female), SEER stage (localized, regional or distant); The greater the OR value, the greater the possibility of dying within one month after surgery; Due to missing data or uncommon classification of SEER stage, cancer sites of brain, larynx and prostate weren't included in the multivariable logistic analysis; Abbreviations: NHW, non-Hispanic white; aOR, adjusted odds ratio; CI, confidence interval.

## Abbreviations

SES: socioeconomic status
SEER: Surveillance, Epidemiology, and End Results
NHW: non-Hispanic white
PSM: propensity score matching
aOR: adjusted odds ratio
CI: confidence interval
