# Supplementary material for: Association between Socioeconomic Status and One-Month Mortality after Surgery in 20 Primary Solid Tumors: a Pan-Cancer Analysis
Source: J Cancer. 2020 Jul 11;11(18):5449–55. doi: 10.7150/jca.46088 (PMC7391197; doi:10.7150/jca.46088)
Supplement: Supplementary file 1 — Supplementary figure and tables. [file jcav11p5449s1.pdf]

Figure S1. Flowchart of patient inclusion and exclusion into the study.

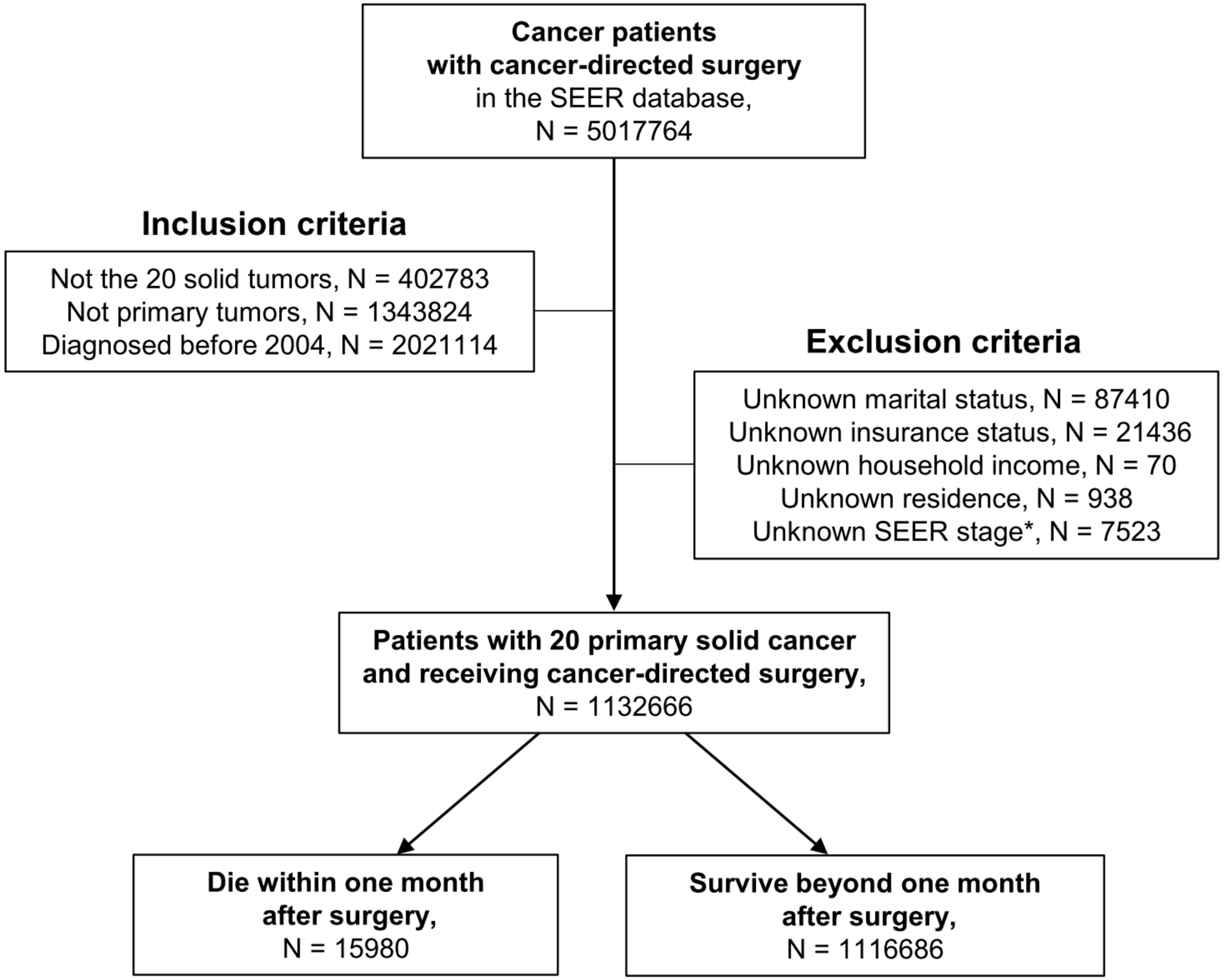

Abbreviations: SEER, Surveillance Epidemiology, and End Results; N, number.

Table S1. Baseline characteristics of included patients by cancer site.

Characteristic		Bladder N=71489	Brain N=25030	Breast N=301471	Cervix N=11919	Colorectum N=161466	Esophagus N=5154	Kidney N=59354	Larynx N=5389	Liver N=9695	Lung N=48460
Group	Die<1 month	1945 (2.7)	1634 (6.5)	432 (0.1)	55 (0.5)	6211 (3.8)	75 (1.5)	664 (1.1)	61 (1.1)	238 (2.5)	1353 (2.8)
	Survive>1 month	69544 (97.3)	23396 (93.5)	301039 (99.9)	11864 (99.5)	155255 (96.2)	5079 (98.5)	58690 (98.9)	5328 (98.9)	9457 (97.5)	47107 (97.2)
Gender	Male	53348 (74.6)	14420 (57.6)	2099 (0.7)	——	82692 (51.2)	4359 (84.6)	36880 (62.1)	4443 (82.4)	6969 (71.9)	23342 (48.2)
	Female	18141 (25.4)	10610 (42.4)	299372 (99.3)	——	78774 (48.8)	795 (15.4)	22474 (37.9)	946 (17.6)	2726 (28.1)	25118 (51.8)
Age		69.7±12.6	48.9±22.2	59.8±13.4	45.7±13.2	64.8±14.1	63.0±10.2	59.0±14.8	63.2±11.6	59.1±15.1	66.3±10.8
Race	NHW	59062 (82.6)	18211 (72.8)	207882 (69.0)	6602 (55.4)	109063 (67.5)	4367 (84.7)	39977 (67.4)	3894 (72.3)	5051 (52.1)	37920 (78.3)
	Minority	12427 (17.4)	6819 (27.2)	93589 (31.0)	5317 (44.6)	52403 (32.5)	787 (15.3)	19377 (32.6)	1495 (27.7)	4644 (47.9)	10540 (21.7)
Marriage	Married	44998 (62.9)	13650 (54.5)	178989 (59.4)	6057 (50.8)	92810 (57.5)	3522 (68.3)	37770 (63.6)	3063 (56.8)	5667 (58.5)	28671 (59.2)
	Unmarried	26491 (37.1)	11380 (45.5)	122482 (40.6)	5862 (49.2)	68656 (42.5)	1632 (31.7)	21584 (36.4)	2326 (43.2)	4028 (41.5)	19789 (40.8)
Insurance	Insured	64188 (89.8)	20120 (80.4)	262923 (87.2)	8236 (69.1)	137162 (84.9)	4603 (89.3)	50704 (85.4)	4075 (75.6)	7579 (78.2)	42407 (87.5)
	Medicaid/ Uninsured	7301 (10.2)	4910 (19.6)	38548 (12.8)	3683 (30.9)	24304 (15.1)	551 (10.7)	8650 (14.6)	1314 (24.4)	2116 (21.8)	6053 (12.5)
Income	Top 50%	34189 (47.8)	12164 (48.6)	146043 (48.4)	5134 (43.1)	71343 (44.2)	2353 (45.7)	26014 (43.8)	2101 (39.0)	5026 (51.8)	22108 (45.6)
	Bottom 50%	37300 (52.2)	12866 (51.4)	155428 (51.6)	6785 (56.9)	90123 (55.8)	2801 (54.3)	33340 (56.2)	3288 (61.0)	4669 (48.2)	26352 (54.4)
Education	Top 50%	34251 (47.9)	12217 (48.8)	150946 (50.1)	5289 (44.4)	73204 (45.3)	2327 (45.1)	26479 (44.6)	2189 (40.6)	5129 (52.9)	21914 (45.2)
	Bottom 50%	37238 (52.1)	12813 (51.2)	150525 (49.9)	6630 (55.6)	88262 (54.7)	2827 (54.9)	32875 (55.4)	3200 (59.4)	4566 (47.1)	26546 (54.8)
Residence	Metropolitan	62567 (87.5)	22336 (89.2)	271252 (90.0)	10746 (90.2)	141038 (87.3)	4416 (85.7)	52023 (87.6)	4503 (83.6)	8958 (92.4)	41842 (86.3)
	Rural	8922 (12.5)	2694 (10.8)	30219 (10.0)	1173 (9.8)	20428 (12.7)	738 (14.3)	7331 (12.4)	886 (16.4)	737 (7.6)	6618 (13.7)
Unemploy-ment	Top 50%	37701 (52.7)	12767 (51.0)	155728 (51.7)	6709 (56.3)	86069 (53.3)	2557 (49.6)	31480 (53.0)	2891 (53.6)	4925 (50.8)	25518 (52.7)
	Bottom 50%	33788 (47.3)	12263 (49.0)	145743 (48.3)	5210 (43.7)	75397 (46.7)	2597 (50.4)	27874 (47.0)	2498 (46.4)	4770 (49.2)	22942 (47.3)
Poverty	Top 50%	35243 (49.3)	12465 (49.8)	153345 (50.9)	6824 (57.3)	87407 (54.1)	2414 (46.8)	32332 (54.5)	3031 (56.2)	4710 (48.6)	25097 (51.8)
	Bottom 50%	36246 (50.7)	12565 (50.2)	148126 (49.1)	5095 (42.7)	74059 (45.9)	2740 (53.2)	27022 (45.5)	2358 (43.8)	4985 (51.4)	23363 (48.2)
SEER stage	Localized	54787 (76.6)	——	198979 (66.0)	8613 (72.3)	72514 (44.9)	1788 (34.7)	43135 (72.7)	——	7228 (74.6)	24201 (49.9)
	Regional	13866 (19.4)	——	92363 (30.6)	2717 (22.8)	64086 (39.7)	2746 (53.3)	11534 (19.4)	——	2039 (21.0)	20068 (41.4)
	Distant	2836 (4.0)	——	10129 (3.4)	589 (4.9)	24866 (15.4)	620 (12.0)	4685 (7.9)	——	428 (4.4)	4191 (8.6)

Characteristic		Melanoma N=62920	Oral cavity N=18419	Ovary N=27371	Pancreas N=11570	Prostate N=136515	Small intestine N=7394	Stomach N=16250	Testis N=15752	Thyroid N=69694	Uterine N=67354
Group	Die<1 month	191 (0.3)	119 (0.6)	707 (2.6)	395 (3.4)	245 (0.2)	280 (3.8)	656 (4.0)	60 (0.4)	159 (0.2)	500 (0.7)
	Survive>1 month	62729 (99.7)	18300 (99.4)	26664 (97.4)	11175 (96.6)	136270 (99.8)	7114 (96.2)	15594 (96.0)	15692 (99.6)	69535 (99.8)	66854 (99.3)
Gender	Male	34901 (55.5)	10942 (59.4)	——	5934 (51.3)	——	3866 (52.3)	9675 (59.5)	——	15509 (22.3)	——
	Female	28019 (44.5)	7477 (40.6)	——	5636 (48.7)	——	3528 (47.7)	6575 (40.5)	——	54185 (77.7)	——
Age		58.0±16.5	60.1±15.4	58.0±15.0	63.6±12.1	62.4±8.4	61.7±13.3	64.7±13.7	34.0±11.8	47.9±15.0	61.0±11.5
Race	NHW	58312 (92.7)	13636 (74.0)	18940 (69.2)	8118 (70.2)	98077 (71.8)	5213 (70.5)	7909 (48.7)	10594 (67.3)	45439 (65.2)	46786 (69.5)
	Minority	4608 (7.3)	4783 (26.0)	8431 (30.8)	3452 (29.8)	38438 (28.2)	2181 (29.5)	8341 (51.3)	5158 (32.7)	24255 (34.8)	20568 (30.5)
Marriage	Married	42463 (67.5)	10721 (58.2)	15023 (54.9)	7496 (64.8)	107997 (79.1)	4649 (62.9)	10293 (63.3)	6795 (43.1)	45275 (65.0)	36875 (54.7)
	Unmarried	20457 (32.5)	7698 (41.8)	12348 (45.1)	4074 (35.2)	28518 (20.9)	2745 (37.1)	5957 (36.7)	8957 (56.9)	24419 (35.0)	30479 (45.3)
Insurance	Insured	58699 (93.3)	15543 (84.4)	23035 (84.2)	10143 (87.7)	128742 (94.3)	6394 (86.5)	13093 (80.6)	12060 (76.6)	61361 (88.0)	57900 (86.0)
	Medicaid/ Uninsured	4221 (6.7)	2876 (15.6)	4336 (15.8)	1427 (12.3)	7773 (5.7)	1000 (13.5)	3157 (19.4)	3692 (23.4)	8333 (12.0)	9454 (14.0)
Income	Top 50%	33354 (53.0)	8632 (46.9)	13428 (49.1)	5697 (49.2)	63637 (46.6)	3400 (46.0)	7468 (46.0)	7799 (49.5)	35523 (51.0)	33119 (49.2)
	Bottom 50%	29566 (47.0)	9787 (53.1)	13943 (50.9)	5873 (50.8)	72878 (53.4)	3994 (54.0)	8782 (54.0)	7953 (50.5)	34171 (49.0)	34235 (50.8)
Education	Top 50%	33960 (54.0)	8684 (47.1)	13763 (50.3)	5794 (50.1)	66241 (48.5)	3595 (48.6)	7633 (47.0)	7874 (50.0)	36025 (51.7)	33602 (49.9)
	Bottom 50%	28960 (46.0)	9735 (52.9)	13608 (49.7)	5776 (49.9)	70274 (51.5)	3799 (51.4)	8617 (53.0)	7878 (50.0)	33669 (48.3)	33752 (50.1)
Residence	Metropolitan	55614 (88.4)	16175 (87.8)	24672 (90.1)	10382 (89.7)	122221 (89.5)	6534 (88.4)	14835 (91.3)	14384 (91.3)	63518 (91.1)	60357 (89.6)
	Rural	7306 (11.6)	2244 (12.2)	2699 (9.9)	1188 (10.3)	14294 (10.5)	860 (11.6)	1415 (8.7)	1368 (8.7)	6176 (8.9)	6997 (10.4)
Unemploy-ment	Top 50%	29269 (46.5)	9486 (51.5)	14221 (52.0)	5822 (50.3)	70170 (51.4)	3663 (49.5)	9127 (56.2)	8177 (51.9)	35309 (50.7)	35186 (52.2)
	Bottom 50%	33651 (53.5)	8933 (48.5)	13150 (48.0)	5748 (49.7)	66345 (48.6)	3731 (50.5)	7123 (43.8)	7575 (48.1)	34385 (49.3)	32168 (47.8)
Poverty	Top 50%	28365 (45.1)	9456 (51.3)	13620 (49.8)	5686 (49.1)	69700 (51.1)	3779 (51.1)	8867 (54.6)	7941 (50.4)	33846 (48.6)	33078 (49.1)
	Bottom 50%	34555 (54.9)	8963 (48.7)	13751 (50.2)	5884 (50.9)	66815 (48.9)	3615 (48.9)	7383 (45.4)	7811 (49.6)	35848 (51.4)	34276 (50.9)
SEER stage	Localized	54339 (86.4)	8907 (48.4)	6983 (25.5)	2171 (18.8)	——	2732 (36.9)	6982 (43.0)	10974 (69.7)	43509 (62.4)	48020 (71.3)
	Regional	7081 (11.3)	7634 (41.4)	3192 (11.7)	7501 (64.8)	——	2979 (40.3)	6864 (42.2)	3018 (19.2)	24201 (34.7)	13557 (20.1)
	Distant	1500 (2.4)	1878 (10.2)	17196 (62.8)	1898 (16.4)	——	1683 (22.8)	2404 (14.8)	1760 (11.2)	1984 (2.8)	5777 (8.6)

Note: All variables were expressed as frequency (percent), except for age as mean ± standard deviation; Some characteristics were not available, such as uncommon classification of SEER stage for cancer sites of brain, larynx and prostate had, and single gender for cancer sites of cervix, ovary, prostate, testis and uterine.

Abbreviations: N, number; NHW, non-Hispanic white; SEER, Surveillance Epidemiology, and End Results.

Table S2. Baseline characteristics of patients after propensity score matching.

Characteristic	Die within one month (N=12718)	Survive beyond one month (N=12718)	*P*	Characteristic	Die within one month (N=12718)	Survive beyond one month (N=12718)	*P*
Tumor site			1.000	Age			0.915
Bladder	1945 (15.3)	1942 (15.3)			74.6 ± 12.6	74.6 ± 12.5	
Breast	432 (3.4)	433 (3.4)		Race			0.013
Colorectum	6211 (48.8)	6211 (48.8)		NHW	9463 (74.4)	9287 (73.0)	
Esophagus	75 (0.6)	75 (0.6)		Minority	3255 (25.6)	3431 (27.0)	
Kidney	664 (5.2)	661 (5.2)		Marriage			<0.001
Liver	238 (1.9)	246 (1.9)		Married	5860 (46.1)	6842 (53.8)	
Lung	1353 (10.6)	1354 (10.6)		Unmarried	6858 (53.9)	5876 (46.2)	
Melanoma	191 (1.5)	189 (1.5)		Insurance			<0.001
Oral cavity	119 (0.9)	119 (0.9)		Insured	10492 (82.5)	11179 (87.9)	
Pancreas	395 (3.1)	398 (3.1)		Medicaid/ Uninsured	2226 (17.5)	1539 (12.1)	
Small Intestine	280 (2.2)	280 (2.2)		Income			<0.001
Stomach	656 (5.2)	653 (5.1)		Top 50%	5114 (40.2)	5873 (46.2)	
Thyroid	159 (1.3)	157 (1.2)		Bottom 50%	7604 (59.8)	6845 (53.8)	
Gender			0.960	Education			<0.001
Male	6894 (54.2)	6899 (54.2)		Top 50%	5307 (41.7)	5958 (46.8)	
Female	5824 (45.8)	5819 (45.8)		Bottom 50%	7411 (58.3)	6760 (53.2)	
Age group			0.999	Residence			<0.001
<50	459 (3.6)	455 (3.58)		Metropolitan	10971 (86.3)	11186 (88.0)	
50–59	1212 (9.5)	1212 (9.53)		Rural	1747 (13.7)	1532 (12.0)	
60–69	2299 (18.1)	2301 (18.09)		Unemployment			<0.001
>69	8748 (68.8)	8750 (68.80)		Top 50%	7099 (55.8)	6803 (53.5)	
SEER stage			0.999	Bottom 50%	5619 (44.2)	5915 (46.5)	
Localized	3699 (29.1)	3699 (29.1)		Poverty			<0.001
Regional	4757 (37.4)	4754 (37.4)		Top 50%	7281 (57.2)	6626 (52.1)	
Distant	4262 (33.5)	4265 (33.5)		Bottom 50%	5437 (42.8)	6092 (47.9)	

Note: Since missing variables were not allowed for matching, we removed cancer sites of brain, cervix, larynx, ovary, prostate, testis and uterine; All variables were expressed as frequency (percent), except for age as mean ± standard deviation.

Abbreviations: N, number; NHW, non-Hispanic white; SEER, Surveillance Epidemiology, and End Results.

Table S3. Multivariate logistic regression analysis after propensity score matching.

Characteristic	aOR	95% CI	*P*
Race (versus NHW)			
Minority	0.842	0.794-0.894	<0.001
Marriage (versus Married)			
Unmarried	1.315	1.250-1.382	<0.001
Insurance (versus Insured)			
Medicaid/Uninsured	1.495	1.389-1.608	<0.001
Income (versus Top 50%)			
Bottom 50%	1.150	1.050-1.261	0.003
Education (versus Top 50%)			
Bottom 50%	1.064	0.988-1.146	0.102
Residence (versus Metropolitan)			
Rural	1.020	0.942-1.105	0.620
Unemployment (versus Bottom 50%)			
Top 50%	0.978	0.923-1.037	0.458
Poverty (versus Bottom 50%)			
Top 50%	1.068	0.986-1.156	0.104

Note: The greater the OR value, the greater the possibility of dying within one month after surgery; Since missing variables were not allowed for matching, we removed cancer sites of brain, cervix, larynx, ovary, prostate, testis and uterine.

Abbreviations: NHW, non-Hispanic white; aOR, adjusted odds ratio; CI, confidence interval.

Table S4. Multivariate logistic regression analysis of association between socioeconomic factors and one-month postoperative mortality by cancer sites.

Characteristic	Race (versus NHW)		Marriage (versus Married)		Insurance (versus Insured)		Income (versus Top 50%)	
	aOR (95% CI)	*P*	aOR (95% CI)	*P*	aOR (95% CI)	*P*	aOR (95% CI)	*P*
Bladder	0.858 (0.752-0.979)	0.023	1.519 (1.373-1.681)	<0.001	1.574 (1.363-1.818)	<0.001	1.041 (0.875-1.239)	0.647
Brain	1.009 (0.887-1.148)	0.891	1.498 (1.345-1.670)	<0.001	1.367 (1.185-1.577)	<0.001	1.127 (0.934-1.359)	0.211
Breast	1.082 (0.866-1.351)	0.488	1.359 (1.100-1.680)	0.004	2.037 (1.598-2.596)	<0.001	1.376 (0.966-1.961)	0.077
Cervix	1.086 (0.599-1.970)	0.785	1.307 (0.720-2.372)	0.379	1.210 (0.636-2.302)	0.561	4.157 (1.595-10.839)	0.004
Colorectum	0.817 (0.766-0.870)	<0.001	1.443 (1.363-1.527)	<0.001	1.511 (1.403-1.627)	<0.001	1.173 (1.064-1.294)	0.001
Esophagus	1.508 (0.827-2.748)	0.180	1.389 (0.844-2.285)	0.196	1.776 (0.885-3.566)	0.106	1.889 (0.862-4.139)	0.112
Kidney	1.127 (0.946-1.343)	0.179	1.534 (1.303-1.806)	<0.001	1.416 (1.140-1.758)	0.002	1.303 (0.976-1.739)	0.073
Larynx	0.995 (0.539-1.836)	0.987	3.167 (1.787-5.610)	<0.001	1.783 (0.968-3.281)	0.063	1.283 (0.504-3.265)	0.601
Liver	1.171 (0.888-1.545)	0.263	0.955 (0.723-1.260)	0.743	1.640 (1.210-2.223)	0.001	1.056 (0.637-1.748)	0.833
Lung	0.892 (0.773-1.029)	0.116	1.231 (1.096-1.383)	<0.001	1.293 (1.094-1.528)	0.003	0.883 (0.719-1.085)	0.237
Melanoma	0.239 (0.103-0.554)	<0.001	1.809 (1.329-2.463)	<0.001	2.606 (1.770-3.838)	<0.001	1.197 (0.694-2.066)	0.518
Oral cavity	0.986 (0.630-1.544)	0.952	1.459 (0.994-2.143)	0.054	1.947 (1.214-3.123)	0.006	1.179 (0.586-2.371)	0.645
Ovary	1.029 (0.858-1.234)	0.757	1.715 (1.461-2.013)	<0.001	1.536 (1.243-1.897)	<0.001	1.007 (0.762-1.332)	0.960
Pancreas	1.030 (0.814-1.302)	0.807	1.272 (1.024-1.581)	0.030	1.585 (1.180-2.130)	0.002	1.208 (0.833-1.751)	0.318
Prostate	0.956 (0.703-1.301)	0.776	1.661 (1.270-2.172)	<0.001	2.367 (1.644-3.407)	<0.001	1.491 (0.916-2.426)	0.108
Small intestine	1.132 (0.845-1.515)	0.406	1.446 (1.108-1.888)	0.007	2.029 (1.444-2.851)	<0.001	0.971 (0.618-1.525)	0.898
Stomach	0.783 (0.660-0.929)	0.005	1.272 (1.068-1.515)	0.007	1.340 (1.095-1.640)	0.005	1.200 (0.871-1.653)	0.264
Testis	1.475 (0.826-2.637)	0.189	2.032 (1.056-3.911)	0.034	1.908 (1.086-3.349)	0.025	1.256 (0.437-3.615)	0.672
Thyroid	1.149 (0.808-1.634)	0.439	1.771 (1.262-2.485)	<0.001	2.007 (1.352-2.978)	<0.001	1.142 (0.618-2.108)	0.672
Uterine	1.082 (0.887-1.320)	0.436	1.555 (1.286-1.880)	<0.001	1.073 (0.837-1.376)	0.577	1.023 (0.737-1.419)	0.893

Characteristic	Education (versus Top 50%)		Residence (versus Metropolitan)		Unemployment (versus Bottom 50%)		Poverty (versus Bottom 50%)	
	**aOR (95% CI)**	***P***	**aOR (95% CI)**	***P***	**aOR (95% CI)**	***P***	**aOR (95% CI)**	***P***
Bladder	1.136 (0.985-1.310)	0.080	1.048 (0.901-1.220)	0.541	1.042 (0.932-1.165)	0.471	0.942 (0.812-1.093)	0.430
Brain	1.253 (1.073-1.464)	0.004	1.026 (0.871-1.209)	0.761	0.973 (0.861-1.101)	0.667	1.061 (0.903-1.246)	0.470
Breast	1.128 (0.848-1.500)	0.408	0.911 (0.672-1.236)	0.549	0.744 (0.593-0.932)	0.010	0.984 (0.723-1.340)	0.920
Cervical	0.328 (0.154-0.696)	0.004	0.353 (0.081-1.539)	0.166	1.041 (0.522-2.076)	0.909	0.561 (0.221-1.428)	0.226
Colorectal	1.030 (0.951-1.115)	0.472	1.026 (0.945-1.114)	0.541	0.973 (0.914-1.036)	0.398	1.073 (0.987-1.168)	0.099
Esophagus	1.095 (0.559-2.142)	0.792	0.728 (0.351-1.507)	0.392	0.833 (0.482-1.438)	0.511	0.919 (0.471-1.792)	0.804
Kidney	0.966 (0.764-1.221)	0.770	0.846 (0.656-1.092)	0.200	0.942 (0.781-1.136)	0.531	1.043 (0.809-1.344)	0.748
Laryngeal	1.535 (0.695-3.389)	0.289	1.735 (0.893-3.370)	0.104	0.797 (0.445-1.426)	0.444	0.645 (0.298-1.397)	0.266
Liver	1.052 (0.702-1.578)	0.805	1.315 (0.825-2.098)	0.249	0.726 (0.527-0.999)	0.049	1.516 (0.969-2.370)	0.068
Lung	1.055 (0.893-1.245)	0.530	1.092 (0.924-1.292)	0.301	1.017 (0.894-1.156)	0.796	1.361 (1.137-1.629)	<0.001
Melanoma	1.525 (0.958-2.426)	0.075	0.916 (0.591-1.418)	0.693	0.875 (0.621-1.234)	0.447	1.109 (0.706-1.742)	0.654
Oral cavity	1.548 (0.869-2.757)	0.138	0.912 (0.525-1.583)	0.743	1.280 (0.824-1.990)	0.273	0.900 (0.499-1.623)	0.725
Ovarian	1.293 (1.032-1.619)	0.026	0.907 (0.697-1.180)	0.466	1.041 (0.865-1.253)	0.673	1.085 (0.848-1.390)	0.516
Pancreat	0.772 (0.573-1.040)	0.089	1.184 (0.848-1.654)	0.321	1.124 (0.881-1.435)	0.347	1.400 (1.004-1.952)	0.047
Prostate	0.865 (0.577-1.298)	0.485	1.316 (0.903-1.917)	0.153	1.170 (0.853-1.604)	0.330	0.905 (0.601-1.363)	0.632
Small Intestine	1.075 (0.746-1.549)	0.699	1.531 (1.067-2.197)	0.021	1.218 (0.910-1.630)	0.184	1.207 (0.812-1.793)	0.353
Stomach	0.947 (0.737-1.217)	0.671	0.897 (0.663-1.213)	0.481	1.024 (0.836-1.254)	0.817	1.196 (0.902-1.584)	0.213
Testicular	1.377 (0.573-3.309)	0.475	0.881 (0.348-2.233)	0.790	1.125 (0.569-2.225)	0.735	0.799 (0.325-1.963)	0.624
Thyroid	1.476 (0.891-2.444)	0.131	1.009 (0.583-1.745)	0.975	0.949 (0.636-1.417)	0.799	0.939 (0.547-1.610)	0.818
Uterine	1.279 (0.979-1.671)	0.071	1.108 (0.821-1.494)	0.503	0.811 (0.651-1.010)	0.061	1.246 (0.929-1.671)	0.142

Note: The greater the OR value, the greater the possibility of dying within one month after surgery; The ORs were adjusted by age (per one year), gender (male or female), SEER stage (localized, regional or distant), except for brain and larynx by age and gender, for prostate by age, for cervix, ovary, testis and uterine by age and SEER stage.

Abbreviations: NHW, non-Hispanic white; aOR, adjusted odds ratio; CI, confidence interval.

Table S5. Multivariate logistic regression analysis of association between socioeconomic factors and one-month postoperative mortality by genders.

Characteristic	Male		Female	
	**aOR (95% CI)**	***P***	**aOR (95% CI)**	***P***
Race (versus NHW)		0.208		0.001
Minority	1.039 (0.979-1.102)		0.909 (0.858-0.964)	
Marriage (versus Married)		<0.001		<0.001
Unmarried	1.551 (1.474-1.631)		1.453 (1.377-1.533)	
Insurance (versus Insured)		<0.001		<0.001
Medicaid/Uninsured	1.522 (1.417-1.634)		1.698 (1.590-1.812)	
Income (versus Top 50%)		0.007		0.016
Bottom 50%	1.133 (1.034-1.242)		1.115 (1.020-1.219)	
Education (versus Top 50%)		0.159		0.003
Bottom 50%	1.055 (0.979-1.137)		1.117 (1.039-1.201)	
Residence (versus Metropolitan)		0.194		0.838
Rural	1.052 (0.975-1.135)		1.008 (0.932-1.091)	
Unemployment (versus Bottom 50%)		0.690		0.054
Top 50%	1.012 (0.954-1.073)		0.945 (0.892-1.001)	
Poverty (versus Bottom 50%)		0.012		0.110
Top 50%	1.106 (1.022-1.196)		1.066 (0.986-1.152)	

Note: The ORs were adjusted by age (per one year) and SEER stage (localized, regional or distant); The greater the OR value, the greater the possibility of dying within one month after surgery; Due to missing data or uncommon classification of SEER stage, cancer sites of brain, larynx and prostate weren't included in the multivariable logistic analysis.

Abbreviations: NHW, non-Hispanic white; aOR, adjusted odds ratio; CI, confidence interval.

Table S6. Multivariate logistic regression analysis of association between socioeconomic factors and one-month postoperative mortality by age groups.

Characteristic	<50		50–59		60-69		>69	
	**aOR (95% CI)**	***P***	**aOR (95% CI)**	***P***	**aOR (95% CI)**	***P***	**aOR (95% CI)**	***P***
Race (versus NHW)		0.511		0.995		0.143		<0.001
Minority	1.058 (0.893-1.254)		1.000 (0.891-1.121)		1.068 (0.978-1.167)		0.841 (0.797-0.887)	
Marriage (versus Married)		<0.001		<0.001		<0.001		<0.001
Unmarried	1.499 (1.265-1.775)		1.593 (1.426-1.779)		1.541 (1.421-1.671)		1.713 (1.638-1.791)	
Insurance (versus Insured)		<0.001		<0.001		<0.001		<0.001
Medicaid/Uninsured	1.740 (1.461-2.073)		1.882 (1.672-2.118)		1.519 (1.374-1.679)		1.345 (1.258-1.439)	
Income (versus Top 50%)		0.445		0.436		0.755		<0.001
Bottom 50%	1.123 (0.833-1.515)		1.082 (0.888-1.318)		1.024 (0.884-1.186)		1.146 (1.060-1.238)	
Education (versus Top 50%)		0.621		0.061		0.012		0.277
Bottom 50%	1.063 (0.834-1.354)		1.162 (0.993-1.360)		1.162 (1.034-1.307)		1.036 (0.972-1.104)	
Residence (versus Metropolitan)		0.124		0.182		0.816		0.807
Rural	1.229 (0.945-1.599)		1.117 (0.949-1.315)		1.014 (0.899-1.144)		0.992 (0.927-1.061)	
Unemployment (versus Bottom 50%)		0.866		0.189		0.173		0.450
Top 50%	0.983 (0.806-1.199)		0.919 (0.811-1.042)		0.937 (0.854-1.029)		1.020 (0.970-1.072)	
Poverty (versus Bottom 50%)		0.727		0.364		<0.001		0.603
Top 50%	1.048 (0.804-1.367)		1.084 (0.911-1.289)		1.302 (1.144-1.481)		1.018 (0.952-1.088)	

Note: The ORs were adjusted by gender (male or female) and SEER stage (localized, regional or distant); The greater the OR value, the greater the possibility of dying within one month after surgery; Due to missing data or uncommon classification of SEER stage, cancer sites of brain, larynx and prostate weren't included in the multivariable logistic analysis.

Abbreviations: NHW, non-Hispanic white; aOR, adjusted odds ratio; CI, confidence interval.

Table S7. Multivariate logistic regression analysis of association between socioeconomic factors and one-month postoperative mortality by SEER stage.

Characteristic	Localized		Regional		Distant	
	**aOR (95% CI)**	***P***	**aOR (95% CI)**	***P***	**aOR (95% CI)**	***P***
Race (versus NHW)		0.153		0.139		0.015
Minority	1.060 (0.979-1.148)		0.949 (0.885-1.017)		0.921 (0.861-0.984)	
Marriage (versus Married)		<0.001		<0.001		<0.001
Unmarried	1.579 (1.474-1.691)		1.422 (1.337-1.512)		1.520 (1.431-1.613)	
Insurance (versus Insured)		<0.001		<0.001		<0.001
Medicaid/Uninsured	1.879 (1.711-2.063)		1.578 (1.455-1.712)		1.408 (1.305-1.520)	
Income (versus Top 50%)		<0.001		<0.001		<0.001
Bottom 50%	1.166 (1.035-1.314)		1.114 (1.001-1.239)		1.089 (0.979-1.211)	
Education (versus Top 50%)		0.006		0.260		0.064
Bottom 50%	1.147 (1.040-1.265)		1.051 (0.964-1.147)		1.084 (0.995-1.181)	
Residence (versus Metropolitan)		0.663		0.053		0.739
Rural	1.023 (0.923-1.133)		1.093 (0.999-1.196)		0.984 (0.898-1.079)	
Unemployment (versus Bottom 50%)		0.247		0.507		0.759
Top 50%	0.956 (0.885-1.032)		0.977 (0.912-1.047)		0.989 (0.924-1.060)	
Poverty (versus Bottom 50%)		0.168		0.029		0.118
Top 50%	1.075 (0.970-1.191)		1.108 (1.010-1.214)		1.077 (0.981-1.182)	

Note: The ORs were adjusted by age (per one year) and gender (male or female); The greater the OR value, the greater the possibility of dying within one month after surgery; Due to missing data or uncommon classification of SEER stage, cancer sites of brain, larynx and prostate weren't included in the multivariable logistic analysis.

Abbreviations: NHW, non-Hispanic white; aOR, adjusted odds ratio; CI, confidence interval.
